# Integration of multiple lineage measurements from the same cell reconstructs parallel tumor evolution

**DOI:** 10.1016/j.xgen.2022.100096

**Published:** 2022-02-09

**Authors:** Lennart Kester, Buys de Barbanson, Anna Lyubimova, Li-Ting Chen, Valérie van der Schrier, Anna Alemany, Dylan Mooijman, Josi Peterson-Maduro, Jarno Drost, Jeroen de Ridder, Alexander van Oudenaarden

**Affiliations:** 1 Oncode Institute, Hubrecht Institute-KNAW (Royal Netherlands Academy of Arts and Sciences), 3584 CT Utrecht, the Netherlands; 2 Oncode Institute, Center for Molecular Medicine, University Medical Center Utrecht, 3584 CX Utrecht, the Netherlands; 3 Oncode Institute, Princess Máxima Center for Pediatric Oncology, 3584 CS Utrecht, the Netherlands

**Keywords:** single-cell whole-genome sequencing, clonal evolution, tumor heterogeneity

## Abstract

Organoid evolution models complemented with integrated single-cell sequencing technology provide a powerful platform to characterize intra-tumor heterogeneity (ITH) and tumor evolution. Here, we conduct a parallel evolution experiment to mimic the tumor evolution process by evolving a colon cancer organoid model over 100 generations, spanning 6 months in time. We use single-cell whole-genome sequencing (WGS) in combination with viral lineage tracing at 12 time points to simultaneously monitor clone size, CNV states, SNV states, and viral lineage barcodes for 1,641 single cells. We integrate these measurements to construct clonal evolution trees with high resolution. We characterize the order of events in which chromosomal aberrations occur and identify aberrations that recur multiple times within the same tumor sub-population. We observe recurrent sequential loss of chromosome 4 after loss of chromosome 18 in four unique tumor clones. SNVs and CNVs identified in our organoid experiments are also frequently reported in colorectal carcinoma samples, and out of 334 patients with chromosome 18 loss in a Memorial Sloan Kettering colorectal cancer cohort, 99 (29.6%) also harbor chromosome 4 loss. Our study reconstructs tumor evolution in a colon cancer organoid model at high resolution, demonstrating an approach to identify potentially clinically relevant genomic aberrations in tumor evolution.

## Highlights

• Multiple independent lineage tracing modalities obtained in thousands of single cells
• This multi-modal tracing allows construction of highly detailed evolutionary trees
• Application to colon carcinoma organoids shows recurrent chromosomal losses
• Sequential loss of chromosomes 18 and 4 correlates with fitness advantage

## Graphical abstract

**Figure:**
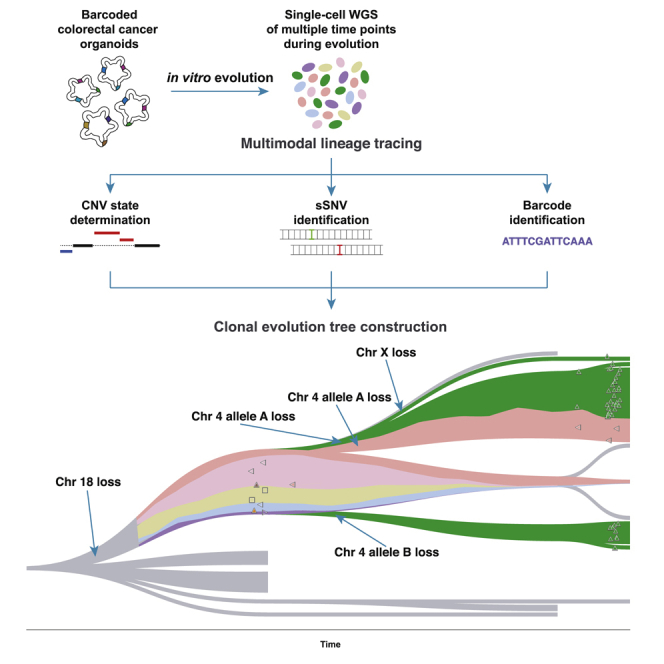

## Teaser

Kester et al. track tumor evolution in colorectal carcinoma organoids using single-cell whole-genome sequencing combined with lineage tracing. Multi-modal tracing based on CNVs, SNVs, and viral lineage barcodes measured simultaneously in single cells enables construction of detailed evolutionary trees and delineation of the molecular events that occur during tumor evolution.

## Introduction

Cancer initiation is the result of cells gradually acquiring genetic alterations due to carcinogenic exposure and DNA replication infidelity.^1^ Some alterations confer a growth advantage resulting in tumor formation in which DNA repair and genome integrity are increasingly ablated, leading to further genetic alterations. As a result, tumor clones arise that exhibit distinct genetic compositions of single-nucleotide variants (SNVs), insertions/deletions (indels), and copy number variants (CNVs). This intra-tumor heterogeneity (ITH)^2^, ^3^, ^4^, ^5^ plays a key role in cancer development. Tumor clones may, for instance, have varying proliferative and metastatic potentials. Furthermore, ITH plays a key role in therapy resistance and the frequent lethal outcome of cancer, since some tumor clones may have intrinsic resistance to therapy.^6^^,^^7^ For this reason, substantial efforts have been made to characterize ITH by mapping the clonal evolution in tumors using whole-genome sequencing (WGS) of the tumor bulk. However, without temporal resolution this approach provides only a limited view oF ITH. It is, for example, difficult to infer the order in which genetic lesions have occurred, and the number of subclones that can be identified is limited.^8^, ^9^, ^10^, ^11^ Although regional sequencing may alleviate some of these issues, it gives only a snapshot of this heterogeneity and does not allow for tracking the evolution of the tumor.^12^

To characterize ITH at high clonal and temporal resolution, genetic alterations in single cells and multiple time points need to be obtained. This is enabled by single-cell DNA sequencing, which was first introduced in 2011^13^ and has since been used to investigate tumor heterogeneity and tumor evolution in many studies.^13^, ^14^, ^15^, ^16^, ^17^, ^18^, ^19^, ^20^ However, most of these studies construct clonal evolution trees exclusively based on either SNVs^18^^,^^20^ or CNVs^13^ and have not utilized the combined information contained in both types of genetic alterations. This combined analysis of CNVs and the SNVs from the same single cell is needed to distinguish recurrent chromosome amplifications or deletions within the same sample. Furthermore, the order of events in which CNVs are established can only be determined with high certainty by combining CNV data with SNV data. Moreover, constructing trees based on multiple independent lineage tracing strategies provides for internal validation by evaluating consistency across multiple independent lineage markers. Most clonal evolution studies based on single-cell analyses have been limited in the number of cells and/or the number of SNVs that are interrogated and do not include multiple time points. A few existing studies do combine SNV and CNV measurements from the same single cells in tumors.^17^^,^^19^ However, these studies rely on a limited number of SNVs and low-resolution CNV data, resulting in shallow clonal evolution trees that lack the resolution to acquire a complete picture of the clonal evolution of these tumors.

In this study, we combine high-resolution CNV data with high-quality SNVs from thousands of single cells to improve the ability to delineate accurate clonal evolution trees. To increase the accuracy of the clonal evolution trees even further, we added a viral barcode-based lineage tracing strategy and combined this with our analyses based on single-cell DNA sequencing-based detection of SNVs and CNVs. This provides three (CNVs, SNVs, and the lineage barcodes) complementary levels of lineage tracing that can be integrated to acquire a complete view of tumor evolution and that allow internal validation of the resulting clonal evolution trees. We moreover introduce a temporal axis to the data by taking multiple time points during clonal evolution.

We utilize a colon carcinoma organoid model, selected as a relevant system for characterizing SNVs and CNVs in ITH and tumor evolution. Colon carcinoma is frequently initiated by mutations in Wnt, epidermal growth factor receptor (EGFR), P53, and transforming growth factor (TGF)-β signaling pathways. Furthermore, colon carcinoma is often associated with chromosomal instability (CIN) resulting in widespread CNVs.^21^ Recent studies have shown that the formation of colon carcinoma can be accurately mimicked *in vitro* using an organoid model.^22^ This organoid model uses CRISPR-Cas9 to induce sequential mutations in *APC*, *TP53*, *KRAS*, and *SMAD4*. *APC*^−/−^ *TP53*^−/−^ *KRAS*^G12D^ *SMAD4*^−/−^ (APKS) organoids morphologically and phenotypically resemble carcinoma-stage colorectal tumors and have pronounced CIN. The CIN results in genetically heterogeneous cultures mimicking the ITH observed in clinical samples of colorectal carcinoma (CRC).^23^ Copy number changes that are found in both the APKS organoids and CRC patients include chromosomes 4, 18, and 8.^24^, ^25^, ^26^, ^27^ Furthermore, chromosome 4 and 18 deletions (Δ4, Δ18) are common in tetraploid colon cancer tumors.^28^

After introduction of the viral barcodes into the organoid model at the beginning of the experiment, the organoids undergo a 26-week period of *in vitro* evolution, during which single-cell DNA sequencing is performed at 12 time points to detect CNVs, SNVs, and viral lineage barcodes. In parallel, the relative amount of each tumor clone was analyzed weekly through bulk sequencing of the lineage barcode. This combination of lineage measurements allows the construction of unprecedentedly detailed clonal evolution trees. Furthermore, the combination of three markers (CNVs, SNVs, and the viral lineage barcodes) provides for internal validation of the constructed trees, as trees constructed based on two markers can be validated by the third. We use this combined approach to construct clonal evolution trees and, from these, characterize the order of events in which chromosomal deletions and amplifications occur and identify chromosomal aberrations that occur multiple times independently within the same cell population. Notably, our clonal evolution trees showed the sequential Δ4 followed by Δ18 in multiple unique tumor clones. This combination of Δ4 and Δ18 provides a strong proliferative advantage in our experiments and is correlated with reduced recurrence-free patient survival in colorectal cancer patients in a Memorial Sloan Kettering Cancer Center (MSKCC) colorectal cancer cohort compared to patients with only a single Δ18. Although this approach highlights the potential relevance of these genome alterations, further clinical studies in additional cohorts are needed to evaluate the clinical implications.

## Results

### Lineage tracing in colon carcinoma organoids reveals clonal dynamics

**Figure 1:**
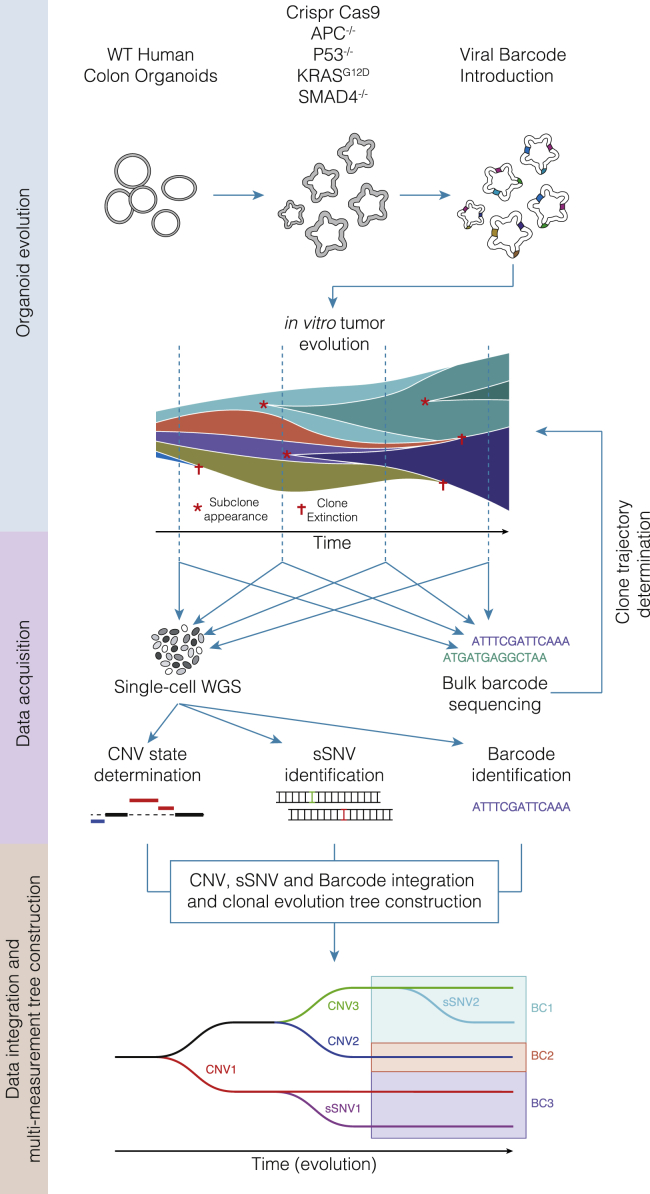
Experimental setup Wild-type human colorectal organoids were transformed using a CRISPR-Cas9-based strategy.^22^ Transformed organoids were transduced with a lentiviral library introducing a lineage barcode. Organoids underwent a 26-week *in vitro* evolution period during which single-cell WGS was performed at regular intervals and culture complexity was assessed weekly. From the single-cell WGS, copy number state, sSNV state, and lineage barcodes were acquired, allowing the construction and validation of highly detailed clonal evolution trees.

To track the clonal evolution of colon carcinoma, we used early-passage human-derived APKS colon organoid cultures in triplicates (hereafter referred to as replicate 1, replicate 2, and replicate 3). After establishment of the organoid line, a viral lineage library with around 60,000 unique barcodes was introduced. The organoids subsequently underwent a 26-week (25 passages) *in vitro* evolution period (Figure 1). The relative abundance of each of the viral barcodes was analyzed weekly through bulk sequencing of the lineage barcode (weeks 4 through 26). By assessing the relative barcode abundance in all three replicates, we observed rapid expansion of a relatively small number of clones (Figure S1). An important question is whether the observed dynamics could be explained by neutral drift in the culture. To exclude this possibility, we performed stochastic simulation of the organoid culture (STAR Methods), taking into account the proliferation rate of the organoids, the number of individual cells at the start of the experiment (start population size), and the number of cells that is left after passaging of the organoids (bottleneck size). We observed that the decrease in entropy in the actual experiment is significantly faster than in the simulations, indicating that these clonal dynamics could not be explained by neutral drift in the culture (Figure S2), indicating there is a selection process underlying the clonal dynamics. Furthermore, we observe expansion of the same clones (as determined through CNVs) in multiple replicates, providing additional support for a strong selective pressure on the organoids.

### High-resolution CNV detection allows identification of 52 unique CNV states

**Figure 2:**
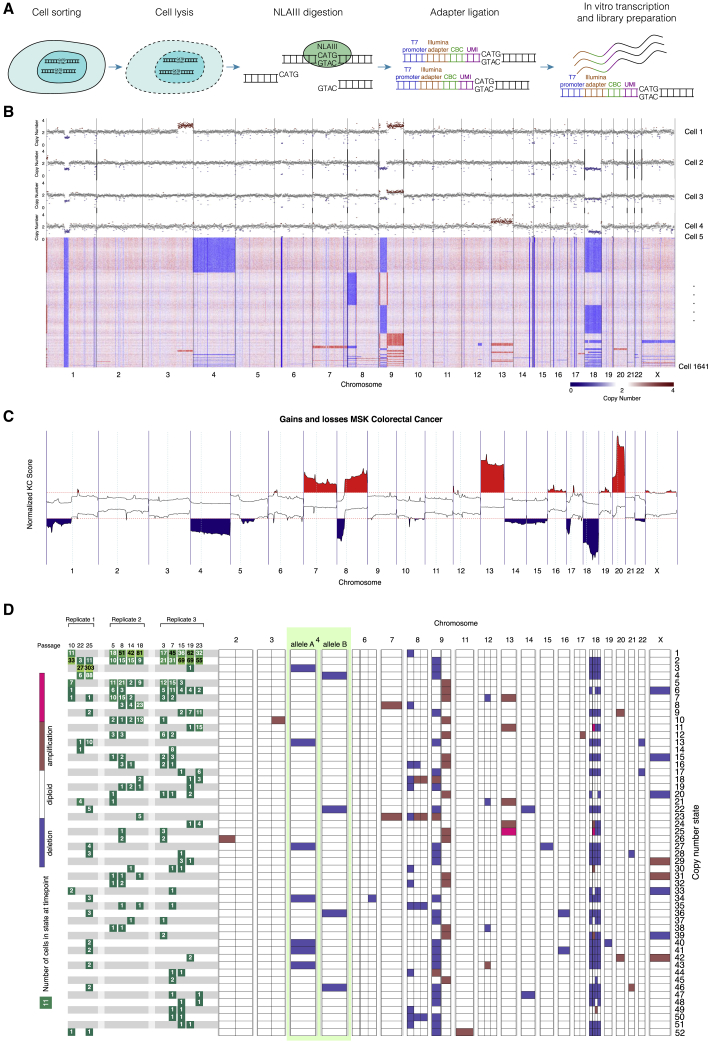
CNV state landscape during clonal evolution (A) Schematic overview of single-cell WGS strategy. (B) CNV profile of the 1,641 single cells from which single-cell WGS data were acquired. (C) Frequently occurring chromosomal aberrations in the Memorial Sloan Kettering Colorectal Cancer dataset. (D) Overview of the 52 unique CNV states identified in the organoids. Each line represents a CNV state; the left part of the figure shows the abundance of the CNV states in the three replicates at the different time points. The right part of the figure shows the deletions and amplification that were detected in each of the CNV states. Chromosome 4 has been split into allele A and allele B.

In parallel to the bulk analysis of the viral lineage barcodes, single cells were harvested at regular intervals and processed for single-cell DNA sequencing using an NLA-III restriction enzyme-based technique^29^^,^^30^ (Figure 2A). Adapters containing unique molecule identifiers (UMIs), allowing quantification of the absolute number of unique molecules in each single cell, are ligated to the NLA-III cut sites, and the molecules are amplified using *in vitro* transcription (IVT) prior to sequencing. After binning the mapped reads using 500 kb bins and filtering of cells with too few reads or fragmented genomes, a total of 1,641 cells with a mean number of 326,000 unique molecules per cell remained (Figure 2B). Copy number profiles were normalized by dividing by the median and multiplying by 2. Dimensionality reduction using principal-component analysis on the median-normalized matrix shows that the cells cluster by replicate and time point, whereas for early time points the cells of the various replicates are more similar (Figure S3). Recurrent break points between regions with different copy numbers were detected by hierarchical clustering followed by circular binary segmentation (STAR Methods). The high resolution and low noise (Figure S4) of the NLA-III restriction enzyme-based technique allows accurate quantification of the CNV profile for each single cell. For instance, we observed multiple unique CNVs affecting chromosome 18, which could not have been detected through bulk WGS (Figure 2D; for example, copy number states 2, 6, and 11).

In parallel to single-cell NLA-III sequencing, we performed standard bulk WGS at the first and last day of the *in vitro* evolution. In these bulk data we observed a full Δ4 in replicate 1 at the end of the experiment. However, the B-allele frequency (BAF) revealed that both alleles were still present, albeit in unequal amounts (Figure S5). This indicated that a fraction of cells had lost one allele of chromosome 4, and the rest of the cells had lost the other allele. To confirm this in the single cells, we first acquired the diplotype of chromosome 4, based on another organoid line derived from the same donor that had completely lost one of the alleles of chromosome 4. This diplotype was then used to assess which allele (if any) of chromosome 4 was lost in each of the single cells. Indeed, in replicate 1 we observed 314 single cells with a Δ4 allele A and 96 cells with a Δ4 allele B. The diplotype for chromosome 18 could also be acquired. Here we observed that all the unique deletions on chromosome 18 concern the same allele. The observed single-cell BAFs of chromosome 4 and 18 are tri-modal, with peaks around 0, 0.5, and 1, indicating the cells were diploid and not tetraploid (Figure S5B). These observations demonstrate that the combination of single-cell NLA-III sequencing and WGS allows allele-specific CNV detection in single cells. Most of the deletions and amplifications observed in the organoid culture are also frequently observed in patients in CRC, emphasizing the relevance of the organoid model for studying colorectal cancer (Figure 2C).

In total we identify 25 unique CNVs across 1,641 single cells, with 52 unique CNV states (we define a CNV state as a genome-wide CNV profile that is shared by at least two single cells; STAR Methods), ranging in size from 434 cells to 3 cells (Figure 2D). In replicate 1 we observe a massive expansion of cells with a Δ4 and a Δ18, whereas in the second (replicate 2) and third (replicate 3) experiment we observe expansion of cells with a Δ8p.

### Construction of high-resolution clonal evolution trees

**Figure 3:**
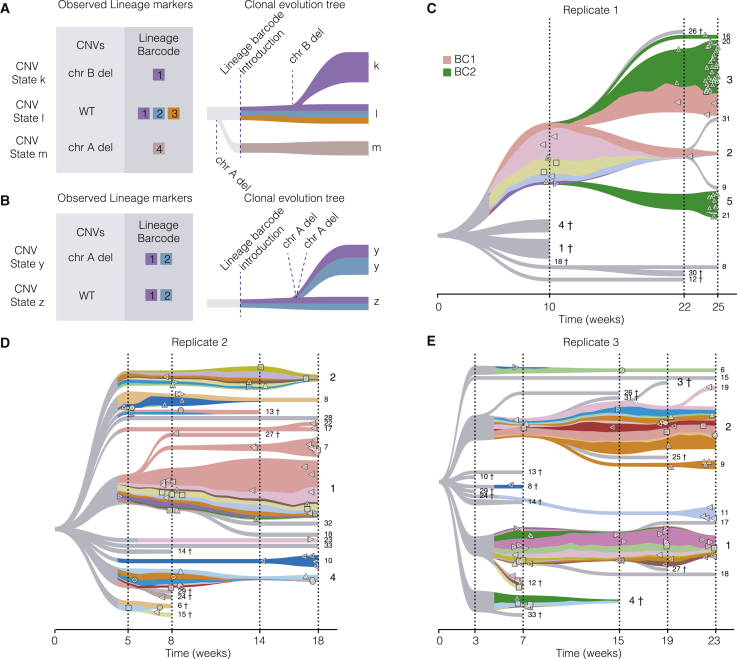
Viral lineage barcodes projected on CNV-based clonal evolution trees (A and B) Examples of CNV events that can be explained by the combination of CNV state and viral lineage barcode information. (C–E) Viral lineage barcodes projected on CNV-based clonal evolution trees for replicates 1 through 3. Numbers on the right side of each tree indicate the CNV state. 十 indicates CNV state has gone extinct.

To construct initial clonal evolution trees based on CNVs, we used the CNV states to create a directed edit distance graph. Since the same CNV state can be present at multiple time points, each time point was added as a separate node in the graph. This enables enforcing temporal consistency (i.e., earlier time points could not be derived from later time points) in the tree construction (STAR Methods). A spanning arborescence was extracted from the directed CNV edit distance graph using Edmonds’ algorithm.^31^ The clonal evolution trees were visualized using ToverBoom (Figures 3C–3E, STAR Methods, Figure S6). The resulting clonal evolution trees indicate the most likely evolutionary trajectories along which the tumor has evolved. The tree for replicate 1, for instance, indicates that CNV state 3 is a descendant of CNV state 2, which is logical considering that CNV state 2 has Δ18 and CNV state 3 has Δ18 and Δ4 (Figure 2D). In conclusion, the high-resolution CNV calling allows construction of detailed clonal evolution trees with a temporal component.

### Integration of CNV states with an independent lineage marker is required for validation of clonal evolution trees

Although the above-described clonal evolution trees derived based on CNV states indicate the most likely evolutionary trajectory, we cannot exclude the possibility that two seemingly related CNV states arose independently, in particular given that copy number changes occur frequently in this genetic background. To disambiguate the relation between two CNV states, we can leverage the information provided by the viral lineage barcode. This is schematically represented in Figures 3A and 3B. For a new CNV state to be introduced during the experiment, both the cells in the new CNV state and their parental cells must be marked by the same viral lineage barcode. For example, the new CNV state *k* arose from the parental CNV state *l* because both states share viral lineage barcode 1 (Figure 3A). On the other hand, CNV states observed in cells that do not share a viral lineage barcode with their putative parent most likely arose prior to the introduction of the lineage markers (illustrated by CNV state *m* in Figure 3A). Similarly, a CNV state that contains cells with multiple lineage markers most likely also arose prior to barcode introduction (CNV state *l* in Figure 3A).

A clone in a certain CNV state harboring multiple lineage markers, which are also present in their inferred parental CNV state (for example, CNV state *y* and CNV state z in Figure 3B), are particularly interesting. In this example, CNV state z and CNV state *y* both harbor viral lineage barcodes 1 and 2, indicating that these CNV states are closely related and arose after lineage marker introduction. Since the viral lineage barcodes mark unique lineages, this implies that the loss of chromosome A occurred twice independently, once in a cell with lineage barcode 1 and once in a cell with lineage barcode 2. An alternative explanation for the observation of CNV states sharing lineage barcodes is that these CNV states were already present at the start of the experiment and that the same lineage barcode was introduced multiple times into cells with these CNV states. However, this is very unlikely due to the large number of barcodes present in the viral lineage library (based on simulations, the probability of two cells in the starting culture receiving the same viral barcode is smaller than 0.0001).

To detect the viral lineage marker in single cells, allowing us to disambiguate and validate clonal evolution trees, we employed an experimental strategy that enriches for reads containing the viral lineage barcode (STAR Methods**,** Figure S7). This allowed us to detect the lineage barcode for 293 of the sequenced single cells. The lineage barcode information can be superimposed on the clonal evolution trees, which allows us to distinguish between the cases described previously (Figures 3A and 3B). Indeed, we observe shared viral lineage barcodes between several CNV states that are descending from each other according to the CNV-based clonal evolution trees. For instance, CNV state 2 (Δ18) and CNV state 3 (Δ18 and Δ4) share a viral lineage barcode, BC1 (Figure 3C). This confirms that during the course of the *in vitro* evolution, a single cell with BC1 belonging to CNV state 2 lost the A allele of chromosome 4, thereby founding CNV state 3. Another shared viral lineage barcode, BC2, was also observed between CNV states 3, 5, 16, 20, and 21, all of which share Δ18. This indicates that, even though this particular viral lineage barcode is not observed in CNV state 2, it must have been present and was most likely not observed due to sampling. Other examples of new CNV states arising during the *in vitro* evolution period include CNV state 7, 17, 22, and 27 from CNV state 1 in replicate 2 (Figure 3D) and CNV state 9 from CNV state 2 in replicate 3 (Figure 3E). Interestingly, in replicate 2, the CNV-based clonal evolution tree suggests that CNV states 17 and 22 arose independently from CNV state 1. Based on the viral lineage barcodes, we can conclude that this is false and that CNV states 17 and 22 descend from CNV state 1. This illustrates the importance of integrating multiple lineage measurements to achieve a more accurate picture of tumor evolution.

Besides observing multiple CNV states sharing the same viral lineage barcode, we also observe multiple viral lineage barcodes within the same CNV state (e.g., CNV states 1, 2, 4, and 6). The most likely explanation for this is that these CNV states were already present at the moment of viral lineage barcode introduction (Figure 3A). The observation that CNV states 1, 2, 4, and 6 were already present at the start of the experiment is confirmed by the fact that these states are all present in multiple replicates. Furthermore, we already observe a subclonal Δ18 (CNV state 2) in the bulk WGS from samples taken at the start of the experiment (Figure S5D).

### Somatic SNVs provide an additional layer of information to increase tree resolution

Somatic SNVs (sSNVs) can be used as lineage markers as they are inherited from one cell to its progeny. Shared sSNVs thus indicate a shared common ancestor, and sSNVs can be used to disambiguate phylogenetic relationships between previously identified CNV states. Whereas copy number alterations are likely to have a fitness effect, most sSNVs are passenger mutations without any effect on the fitness of the cells, thus providing a lineage marker that is less affected by selection. Moreover, unlike viral lineage markers, sSNVs accumulate throughout time and therefore provide lineage marking of clones that initiate during the evolution experiment. For these reasons, in addition to the viral barcodes and copy number profiles, we assess sSNVs within the cells of all three replicates. This additional layer of information allows us to identify additional heterogeneity within the population of cells with the same copy number state, verify edges of the inferred lineage trees based on the copy numbers, and identify copy number aberration events that occurred multiple times.

sSNVs called from single-cell sequencing data suffer from high numbers of false positive calls. To identify reliable sSNVs, we therefore trained a random forest (RF) classifier on the somatic mutations that could be verified in the bulk library and used the trained classifier to identify reliable sSNVs (STAR Methods). Every variant that passed the RF filter is phased to at least one heterozygous germline variant or otherwise discarded. Positive variant calls are identified by presence of the alternative allele among all sequence reads for the position within a cell. Negative variant calls are identified by presence of the reference allele in phase with the germline variant found to be linked with the alternative allele.^32^^,^^33^ This procedure allowed us to extract 106 high-quality sSNVs from all cells used for tree inference.

**Figure 4:**
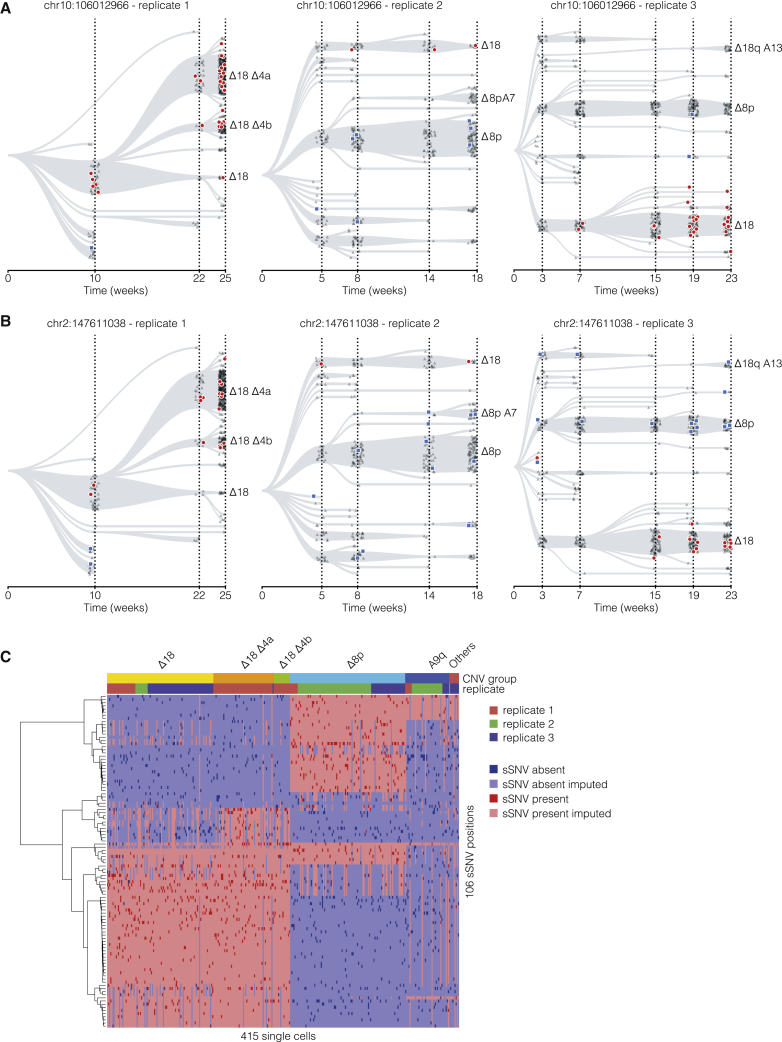
sSNVs projected on CNV-based clonal evolution trees (A and B) The presence of one sSNV projected onto CNV trees of three replicates. Each marker indicates a single cell, and its color and shape indicate whether the alternative allele is detected (red, circle) or the reference allele (blue, square) is detected or the sSNV is not covered (gray, triangle). (C) Single-cell sSNV matrix. Cells are grouped based on their copy number state.

sSNVs can be overlaid on the lineage trees inferred from the copy number calls. Figures 4A and 4B show this for two example sSNVs across all three replicates. The sSNV can be present (red markers), absent (the reference allele is detected, blue markers), or undetermined (insufficient coverage to be detected, gray markers). This example shows that subclones of the Δ18 clone always carry the variant, whereas the Δ8p clone including subclones do not. This confirms that there is strong association of these two SNVs to clones with a similar copy number profile.

By clustering based on all detected sSNVs, the cells separate in two main groups. The first group of cells is characterized by Δ18, whereas the second predominantly carries a Δ8p (Figure 4C). Most variants are detected in multiple replicates, which indicates that the variant was likely present before the replicates were separated.

### Integrating sSNVs with CNVs in single cells suggests parallel evolution of copy number states

**Figure 5:**
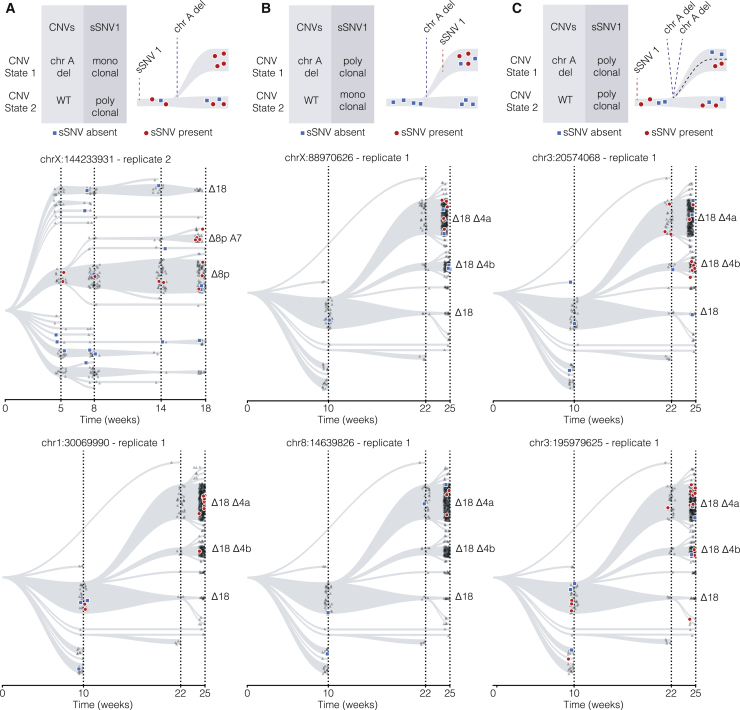
Three classes of sSNVs at the branching point of two copy number states Schematic representations of classes 1–3 on the top row. The two rows below show identified instances of each class. Each marker indicates a single cell. Its color and shape indicate whether the alternative allele (red, circle) or the reference allele (blue, square) is detected or the sSNV is not covered (gray, triangle). (A) In class 1, the copy number aberration occurs after the sSNV appearance. (B) In class 2, the copy number aberration occurs before the sSNV appearance. (C) Class 3 is an example of a parallel evolution event, in which a sSNV is followed by two independent copy number aberrations.

In addition to the strong co-segregation of copy number state and somatic variants (Figure 4C), we also observe more complex relations between the two lineage markers. We define three classes of sSNVs at the branching point of two copy number states (Figure 5).

In the first class, a copy number aberration occurs after the sSNV. This would be consistent with a situation in which, at an early time point, a clone is marked by an sSNV, and after the CNV-induced bifurcation, the newly derived clone only contains cells carrying the alternative allele (Figure 5A, top). We find examples of this first class on chromosome 8 and chromosome 18 for replicates 2 and 1, respectively (Figure 5A, bottom).

In the second class, an sSNV is introduced after a CNV is created. Here, at early time points the clone does not contain any cells with the sSNV. After branching into a new copy number state, the sSNV is exclusively observed for the cells in the new CNV state, indicating it must have been introduced after the acquisition of the CNV (Figure 5B, top). This class of sSNVs can be used to verify edges in the copy number tree similar to the viral lineage markers. Examples of the second class are shown for replicate 1 occurring within Δ18 subclones (Figure 5B, bottom).

In the third and most interesting class, the same CNV occurs twice independently. This situation would be consistent with a clone in a single CNV state that contains cells both with and without an sSNV at an early time point. If after the introduction of a CNV the new clone also contains cells with and without the sSNV, this must mean that the copy number aberration must have occurred at least twice: once in the clone with the sSNV and once in the clone without the sSNV (Figure 5C, top). An example for such a parallel evolution event can be found in multiple variants that show both the reference and mutated alleles in the Δ18 state and the Δ18Δ4A state (Figure 5C, bottom). The same holds for the Δ18 to Δ18Δ4B state (Figure 5C, bottom).

Accurately identifying the first two classes is challenging, because it is always possible that the presence or absence of a particular sSNV is not detected because of drop-outs in the single-cell data. However, distinguishing the third class from the first two classes is less vulnerable to sampling errors. If both the presence and absence of an sSNV are detected before and after a CNV is initiated, it rules out the first and second scenario, in particular when this is supported by several sSNVs. Taken together, an integrated analysis of how sSNVs segregated between copy number states suggests that these copy number states can arise multiple times independently.

### Δ18 followed by Δ4

The most highly abundant and fastest growing clone across the three replicates was characterized by a combination of Δ18 and Δ4. Although we did observe 495 cells with Δ18 and without Δ4 (30.2%), divided over 20 CNV states, we did not observe any cells with Δ4 and without Δ18, suggesting that Δ4 results in a proliferative advantage only in the presence of Δ18. This combined Δ18 and Δ4 was detected in a total of 449 single cells (27.3%), divided over 11 CNV states. The probability of this co-occurrence of Δ18 and Δ4 to occur by chance is very small (hypergeometric test p value < 1e^−100^) To see if there is any evidence that supports this hypothesis in clinical samples, we turned to a colorectal cancer cohort from MSKCC.^34^ The MSKCC data show that in colorectal cancer patients chromosomes 18 and 4 are frequently lost (32.5% and 13.8% show log copy ratio < −0.4 for chromosome 18 and chromosome 4, respectively) (Figure 2C).

**Figure 6:**
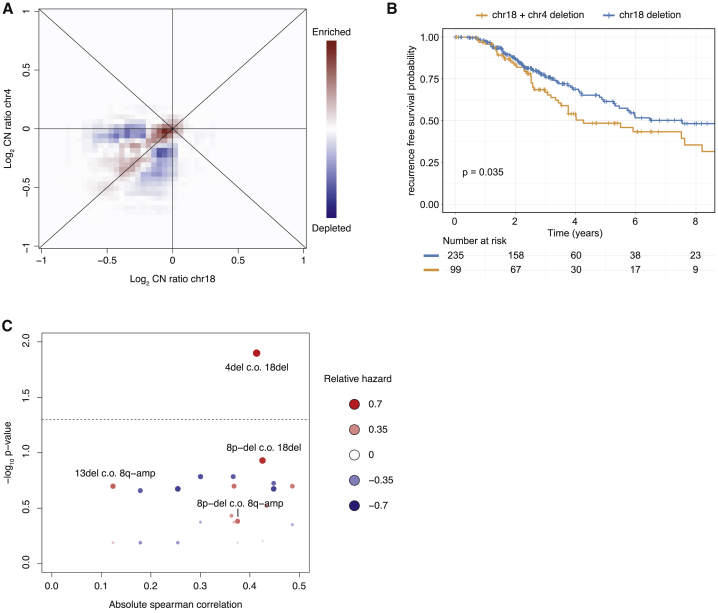
Loss of chromosome 18 followed by loss of chromosome 4 is correlated with reduced survival in the MSKCC patient cohort (A) Relation between Δ18 and Δ4 within the MSKCC patient cohort. Red and blue shading indicate enrichment and depletion compared to a random background distribution. (B) Kaplan-Meier curve comparing survival between patient with Δ18 and Δ4 compared to patient withΔ18 and without Δ4. (C) Absolute correlation between chromosomal alterations compared to the p value for the difference in hazard for the priming event alone compared to the priming event and the conditional event. Shading and size of the points indicate direction and magnitude of the difference in hazard.

However, tumors with a lower copy number ratio for chromosome 4 than for chromosome 18 occur less frequently in the same patient than can be expected based on the chromosome 18 and chromosome 4 copy ratios; in only 27.7% of the tumors the copy ratio for chromosome 18 is lower than chromosome 4 (p value < 0.00001, based on a permutation strategy, STAR Methods, Figure 6A). Therefore, there is an enrichment for tumors in which the copy number ratio for chromosome 18 is lower than for chromosome 4 (72.3% of tumors have lower copy ratio for chromosome 18 than chromosome 4), indicating that most often Δ18 occurs prior to Δ4 (based on a permutation strategy, p value < 0.00001). Strikingly, this is in line with the order of events we observe in the APKS organoid cultures.

We also find that in the MSKCC cohort a combination of Δ18 and Δ4 is correlated with reduced recurrence-free survival compared to patients with Δ18 and without Δ4 (log rank p value < 0.035) (Figure 6B). This suggests that Δ4 in the context of a prior Δ18 is correlated with reduced survival compared to either deletion on its own. To investigate this in a more systematic manner, we investigated all possible combinations of two chromosomal deletions and/or amplifications. We define a “priming event,” which is the first aberration, and a “conditional event,” which is the second aberration in the context of a particular priming event. We then compared the absolute correlation between the copy ratios for any given pair of priming and conditional events to the hazard ratio of the conditional event over the priming event alone (Figure 6C). This analysis finds that in the MSKCC colorectal cancer cohort, Δ18 as priming event followed by Δ4 as conditional event has the highest hazard ratio of all possible conditional events (Cox proportional hazard ratio 0.78, Benjamini-Hochberg corrected p value = 0.012).

These analyses highlight the relevance of our organoid system as a model for colorectal cancer that enables insight into clonal heterogeneity and ordering of mutational events with clinical relevance. Additionally, based on the observations in the organoids, we find that Δ4 conditional on Δ18 is correlated with reduced recurrence-free patient survival. To our knowledge, this is the first example of a conditional chromosomal aberration correlated with reduced survival compared to the corresponding single aberrations.

## Discussion

Delineating the clonal evolution trajectory through which a tumor is formed is pivotal to the understanding of tumor biology. Since every cell inside a tumor is unique, this requires an approach with single-cell resolution. Here, we use single-cell WGS in combination with viral lineage tracing to acquire CNV states, SNV states, and viral lineage barcodes for 1,641 single cells. Almost all of the CNVs identified in the organoids also frequently occur in CRC samples, indicating that the organoids are a valid and valuable model for CRC. We identified 52 unique CNV states in the organoids based on analyses of CNVs in the single cells. From the 52 CNV states, we derived highly detailed clonal evolution trees, which could in turn be internally validated based on the viral lineage markers and the SNVs. This internal validation is only possible due to the multiple independent lineage markers simultaneously.

Our analyses of the clonal evolution trees identified specific CNVs that occurred in multiple independent events in the organoid cultures. The most frequent event observed was Δ4, in at least four independent events. The frequent Δ4 suggests that this event provides the organoids with a proliferative advantage. Indeed, the viral lineage barcodes showed that the clones that lost chromosome 4 expanded during the *in vitro* evolution period.

Clustering of the SNVs identified two main groups, which completely overlap with the two main CNV clones in the data, the Δ18 group and the Δ8p group. However, more detailed interrogation of the SNVs identified several SNVs that can only be explained by multiple occurrences of a specific CNV. This confirms the observation that Δ4 occurred multiple times during the *in vitro* evolution period.

In our data, Δ4 was observed only in the context of Δ18. This suggests that the order of the chromosomal aberrations is in this case important for progression. Analysis of patient data from the MSKCC colorectal cancer dataset found that, in patients, Δ4 also very frequently occurs in the context of Δ18. Furthermore, the combination of Δ18 and Δ4 results in a higher mortality than a Δ18 alone. Strikingly, a further systematic exploration of context-dependent deletions or amplifications in a MSKCC colon carcinoma dataset found that only the conditional Δ4 in the context of Δ18 is correlated with reduced patient recurrence-free survival in comparison to the initial amplification or deletion alone. This indicates that, similar to mutations, the combination of chromosomal aberrations influences aggressiveness of a tumor.

### Limitations of the study

Due to the labor-intensive nature of the organoid culturing experiment, only three replicates have been generated for experiments in this study. For one of our main findings, we observe the sequential Δ18 and Δ4 in only one of the three replicates. However, we also found similar observations in clinical samples. Further studies are needed to estimate the probability of these chromosome loss events in parallel evolution experiments. For this, studies with a larger number of of parallel evolution experiments would be useful, for example, by automating the culturing procedures. This would also allow studies of the mechanistic consequences of specific mutations and copy number changes.

Although we provide an initial examination of the frequency of Δ18 and Δ4 in a single clinical cohort from the MSKCC study, there are limitations. The MSKCC colorectal cancer cohort was generated using the MSK-IMPACT targeted sequencing panel, and therefore the resolution of the copy number variations is limited. Higher-resolution datasets might provide additional insight on segmental chromosomal aberrations. Further studies in additional cohorts are needed to determine potential clinical relevance and confirm the observed correlation with patient survival.

There also are limitations in the sensitivity of current methods for detecting clones based on somatic mutations and copy number profiles. In our study, the copy number profiles have a resolution of about 500 kb, and we were not able to accurately resolve features that are smaller for all cells. Furthermore, phasing of somatic SNVs is not always possible due to the lack of heterozygous germline SNVs in the vicinity of the somatic SNVs. Future studies that improve the sensitivity of detection for somatic mutations and copy number profiles would increase the resolution and therefore could allow construction of even more detailed evolutionary trees. More detailed evolutionary trees allow for more precise understanding of the evolution of a tumor. One currently feasible approach for this would be to use long-read WGS, in combination with a restriction enzyme that cuts less frequently, to increase the number of sSNVs that can be phased to a heterozygous germline variant.

Furthermore, it is likely that the initial population of cells for each replicate is not a pure clonal population. Our observation of shared somatic mutations between replicates suggests that some heterogeneity is present in the initial cultures. At the start of an experiment, it is therefore possible that a replicate is primed with clones that already contain beneficial variants. Previous experiments have shown that even organoids grown from a single cell still contain subclonal aberrations.^34^ Detecting multiple lineage markers in the same cell allows distinguishing between pre-existing mutations and mutations that occurred during the evolution experiment. For example, when a particular sSNV is present in two distinct copy number states, the associated aberration is likely to have occurred during the evolution experiment.

In conclusion, we constructed highly structured clonal evolution trees based on three independent lineage measurements acquired through single-cell WGS of CRC organoids. The lineage measurements identified four independent events in which chromosome 4 was lost in the context of Δ18. This combined Δ18 and Δ4, identified in the organoids, was found to be correlated with reduced survival in a single study of patients with CRC.

## STAR★Methods

### Key resources table

**Table:** 

REAGENT or RESOURCE	SOURCE	IDENTIFIER
**Bacterial and virus strains**		

pCDH lentivector	System Bioscience	CD811A-1
Stable Competent E. Coli cells	NEB	Cat# C3040
pPACKH1 Lentivector Packaging Kit	System Bioscience	Cat# LV500A-1

**Chemicals, peptides, and recombinant proteins**		

NsiI-HF	NEB	Cat# R3127S
Asc1-HF	NEB	Cat# R0558S
T4 DNA Ligase	NEB	Cat# M0202T
QIAGEN Protease	QIAGEN	Cat# 19157
ExoSap	Thermo fisher scientific	Cat# 78205.10.ML
NlaIII	NEB	Cat# R0125S
B27	Thermo fisher scientific	Cat# 17504044
DMEM/F12 medium	Thermo fisher scientific	Cat# 12634010
nicotinamide	Sigma Aldrich	Cat# 72340
N-acetylcysteine	Sigma Aldrich	Cat# A0737
A83-01	Tocris	Cat# 2939
SB202190	Sigma Aldrich	Cat# 7067
Gefitinib	Selleck Chemicals	Cat# ZD1839
Matrigel	Corning	Cat# 354234
Polybrene	Sigma Aldrich	Cat# TR-1003
PEI transfection reagen	Polysciences	Cat# 26406
Puromycin	Sigma Aldrich	Cat# P9620

**Critical commercial assays**		

NEBNext High fidelity PCR mix	NEB	Cat# M0544
AMPure DNA beads	Beckman	Product # A63881

**Deposited data**		

MSKCC dataset	European variation archive	PRJEB23844
Single cell whole genome data, generated in this study	This study	Sequence read archive PRJNA645018

**Experimental models: Cell lines**		

Organoid lines	Drost et al. (2015)^22^; Clevers group, Hubrecht institue	N/A

**Oligonucleotides**		

Viral library barcode PCR primers	This study	Table S1
Truseq small RNA library PCR primers	Illumina	RS-200-0012

**Software and algorithms**		

Burrows Wheeler Aligner	Li and Durbin, 2009^35^	http://bio-bwa.sourceforge.net/
GATKBaseRecalibrator	Van der Auwera et al., 2013^36^	https://gatk.broadinstitute.org/
Mutect	Cibulskis et al., 2013^37^	https://gatk.broadinstitute.org/
Single cell whole genome data processing	This paper	https://doi.org/10.5281/zenodo.5844399
Cutadapt	Martin, 2011^38^	https://github.com/marcelm/cutadapt
ToverBoom	This paper	https://doi.org/10.5281/zenodo.5844342
SciCloneFitIO	This paper	https://github.com/zztin/siCloneFitIO

### Resource availability

#### Lead contact

Further information and requests for resources and reagents should be directed to and will be fulfilled by the lead contact, Alexander van Oudenaarden (a.vanoudenaarden@hubrecht.eu).

#### Materials availability

This study did not generate new unique reagents.

### Experimental model and subject details

#### APKS Organoid culturing

APKS organoids were acquired from the Clevers lab after establishment as described in Drost et al.^22^ Organoid culturing was done as described before in Drost et al.^22^ Culture medium contains advanced DMEM/F12 medium (Thermo fisher scientific) including 1x B27 (Thermo fisher scientific), 10 mM nicotinamide (Sigma-Aldrich), 1.25 mM N-acetylcysteine (Sigma-Aldrich), 500 nM A83-01 (Tocris), 3 uM SB202190 (Sigma-Aldrich) 1 mM gefitinib (Selleck Chemicals). After viral transduction all organoid lines were continuously cultured with 1ug/mL puromycin to make sure that the viral barcode was maintained. Organoids cultures were split weekly by mechanical disruption. Before mechanical disruption, all organoid wells were mixed to ensure all clones were equally distributed. Organoids were mechanically disrupted and ∼300,000 cells were seeded in matrigel (Corning) to maintain the culture. The number of seeded cells exceeds the number of unique barcodes ensuring the seeded population is an appropriate reflection of the pre-split culture. Excess cells were used for DNA extraction and subsequent viral library complexity assessment.

#### Viral library construction

The viral construct was created using the pCDH lentivector CD811A-1 (System Bioscience) in which a GFP was inserted under control of the PGK promotor and a puromycin resistance cassette was inserted under control of the Eef1a promotor. NsiI and AscI restriction sites were inserted in the 5′ UTR of the GFP gene using inverse PCR. The barcode insert was created using a 80bp primer containing the barcode (consisting of 4 stretches of 5 random nucleotides interspersed by A’s) flanked by M13 forward and reverse sequences and restriction sites for Nsi1 and Asc1 (ATGCATGCATTTGTAAAACGACGGCCAGTNNNNNTNNNNNTNNNNNTNNNNNTCACACAGGAAACAGCTATGAGGCGCGCC) and made double stranded using a complementary primer and Klenow fragment (NEB). The insert was subsequently digested with NsiI-HF and Asc1-HF (NEB), to create the right overhangs for ligation into the plasmid. Plasmid was linearized using NsiI-HF and AscI-HF and barcode insert was ligated using T4 DNA Ligase (NEB). Ligated plasmid was transformed into Stable Competent *E. coli* cells (C3040 NEB) and 30.000 colonies were harvested from which plasmids were extracted.

#### Virus generation

HEK293T cells were transfected with 1.2 μg of each of the pPACKH1 Lentivector Packaging Kit (System Bioscience) plasmids and 15.6 μg of the barcoded plasmid library (described above) using PEI transfection (polysciences). Viral particles were harvested 24 h later and concentrated using ultracentrifugation.

### Method details

#### Viral library complexity assessment

8 replicates of 1 μg of viral library were amplified using NEBNext High fidelity PCR mix (NEB) for 10 cycles with barcoded PCR forward primer 1 and PCR reverse primer 1 (Table S1). Illumina sequencing libraries were generated through 5 additional cycles of PCR with Illumina Truseq small RNA library PCR primers. The viral library was sequenced on a NextSeq500 using 2x75bp paired end sequencing. Barcode sequences were merged if they were within hamming distance 2 from each other, merging them into the most abundant of the two, while taking into account sequencing quality.

#### APKS organoid transduction

APKS organoids were trypsinized for 10 min at 37°C. Trypsinized organoids were resuspended in 50 μl medium containing 2x polybrene (Sigma Aldrich) upon which 50 μl of virus mix was added and incubated for 6 h at 37C. After incubation cells were washed and resuspended in 120 μl of Matrigel. Cells were seeded into two wells of a 24 well culturing plate.

#### DNA extraction, barcode amplification and barcode sequencing

For each organoid line DNA was harvested weekly. Cell lysis was performed overnight at 50°C using 0.05 units of QIAGEN Protease in 10 mM Tris pH 7 in a total volume of 1 mL. All samples were split into two and DNA was extracted using phenol/chloroform extraction followed by AMPure DNA bead clean-up (Beckman). Viral barcodes were amplified using a two-step PCR strategy.^39^. All PCRs were done in 96 well plates, using the 96 barcoded forward primers (1 primer per well, Table S1) in combination with a mix of 5 reverse primers. The 5 reverse primers are identical except for a small (0, 1, 2, 3, or 4 base insertion), which ensures high complexity of the libraries, required for sequencing. First, for both replicates of a sample 5 cycles of PCR were performed on 500ng of genomic DNA using NEBnext High Fidelity PCR master mix (NEB) and barcoded primers containing a Unique Molecule Identifier (UMI) (Table S1). After PCR, excess primers were digested using ExoSap (Thermo Fisher Scientific) to prevent UMI replacement during later stages of amplification. After ExoSap treatment PCR reactions were cleaned up using AMPure beads and another 25 cycle PCR was performed using Illumina Truseq small RNA library PCR primers. Libraries were sequenced on Illumina NextSeq 500 using 2x75 bp paired end sequencing. DNA reads were mapped to an artificial reference genome containing 30,190 viral genomes, each with their own unique barcode. Only reads that mapped uniquely to a single viral barcode were considered for further analysis. Library PCR duplicates (based on UMI sharing) were removed. To estimate barcode frequency for each individual time point we used the approach described in,^39^ which uses a Bayesian model to infer the frequency of the barcode in the original culture through the number of reads sequenced in the two replicates from that time point.

#### Whole genome sequencing and bulk variant calling

At passage 4 and passage 21 WGS was performed on the APKS organoids. At passage 4 a mix of DNA from the three replicates was used, while at passage 21 each replicate was sequenced individually. For this DNA was isolated from cells that were left over after passaging the culture. Library preparation and whole genome sequencing was performed at Macrogen using Illumina TruSeq DNA PCR free library preparation and sequenced on a HiSeq 10X with 2 × 150 bp paired end sequencing. Reads were aligned to GRCh38 using Burrows Wheeler Aligner v0.7.14 mapping tool with settings ‘bwa mem –M’^35^. Duplicate reads were marked using Sambamba (version 0.6.6) dedup. Base Quality Score Recalibration was done using GATKBaseRecalibrator v3.7.^36^ Somatic variants were detected using Mutect 2.2.^37^

#### Single Cell Whole Genome Sequencing

Cells were sorted into 384-well plates with 5 μL of mineral oil (Sigma-Aldrich). After sorting, cells can be stored at −20°C. 500 nL of lysis mix (0.0005 u QIAGEN Protease in NEB Buffer 4) was added to each well and lysis was performed at 55°C overnight followed by heat inactivation for 20 min at 75°C and for 5 min at 80°C. 500nl of Restriction Enzyme mix (0.5 u NlaIII in NEB Cutsmart buffer) was added to each well and restriction was performed for 3 h at 37°C followed by heat inactivation for 20 min at 65°C. 100 nL of 1 uM barcoded double stranded NlaIII adaptor was added to each well. 1100 μL of Ligation mix (200 u T4 DNA Ligase in 1x T4 DNA Ligase buffer supplemented with 3 mM ATP) was added to each well and ligation was performed overnight at 16°C. After ligation, single cells were pooled and library preparation was performed as described in Muraro et al.^40^ Libraries were sequenced on an Illumina Nextseq500 with 2 × 75 bp paired end sequencing or on a HiSeq 10X with 2 × 150 bp paired end sequencing.

#### Single cell whole genome data processing

Sequencing data were analyzed through custom *snakemake* workflows (*Python* v3.6), which are available at https://github.com/BuysDB/SingleCellMultiOmics/tree/master/singlecellmultiomics/snakemake_workflows/nlaIII

The UMI and cell barcode were extracted and trimmed from read 1 of the read pair and the 6bp random hexamer was trimmed from read 2. From the resulting trimmed reads, only those starting with the NlaIII recognition sequence CATG were kept. Additionally, adapters were trimmed using cutadapt.^38^ The trimmed reads were mapped to hg38 using BWA 0.7.16a-r1181. Next, the mapping location and strand of the NlaIII recognition sequence in combination with the UMI sequence and cell barcode was used as a unique molecular identifier. This step associates reads to unique molecules in order to deduplicate reads to reduce amplification biases and is used to extract a consensus sequence for each molecule. The consensus base calls are used to genotype germline and somatic SNVs

In order to remove non-uniquely mapping reads, the reference genome was digested in-silico using the NlaIII cut site. For each NlaIII cut site the two flanking fragments were determined for sequences up to 69 bases in length. These fragments were mapped back to the hg38 reference. For each site multi-mapping fragments were recorded. Only molecules mapping to uniquely mappable sites according to the in-silico digestion were kept for copy number analysis. For each cell, molecules were binned in 500kb bins. Bins with fewer than 3000 unique cut sites are considered to have poor mappability and were excluded from the analysis. Due to unavailability of wild-type WGS single cell libraries the copy number profiles could not be normalized against a reference profile. Instead count data was median normalized for each cell and multiplied by 2, resulting in a median copy number of 2 for every cell. Next, we carried out GC bias correction by performing a LOESS regression for the copy number profile of each cell. The corrected values were clipped to a maximum copy number of 4, to mitigate inflated noise at high copy numbers. We find that, even after the rigorous data processing described above, we do not obtain a reliable copy number profile for all cells, these profiles might be caused by cell division or a cell lysis-induced artifact. To filter cells with an unreliable copy number profile we trained a random forest classifier. Training labels were obtained by k-means clustering (k = 12) the cells in UMAP 2D space and manually identifying the cluster which predominantly contains cells with unreliable copy number profiles. The final classifier was applied on the total matrix and all cells with a posterior > 0.99 for the noisy cluster were discarded. The out-of-bag classification score of the random forest was 0.985.

#### Copy number segmentation and state definition

Before copy number segmentation, cells were clustered using Ward’s hierarchical clustering on the Euclidean distance. The number of clusters were set based on the maximum silhouette score, but to ensure conservative (tight) clusters, overclustering was manually enforced for certain large clusters. For each resulting cluster of cells, the mean copy number was calculated per bin and copy number segments were detected using circular binary segmentation^41^ with p = 0.05 and 10,000 shuffles. Segment calls with a mean absolute difference of smaller than 0.6 were rejected. For each cell, the median for each segment was calculated and rounded to the nearest integer.

Segments with variance higher than 0.025 across all cells, which in practice turned out to be small genomic segments with hard to resolve copy numbers, were rejected to prevent those small segments from majorly influencing lineage tree inference. To obtain diplotypes for both chromosome 4 and 18, data from a bulk sample (AP1-P23) derived from the P11N line was leveraged, which contains a complete and clonal loss of both chromosome 4 and 18. For each heterozygous gSNV, the allele with a BAF of 1 is assigned to allele B and the allele with a BAF of 0 to allele A. The A and B allele-frequencies were determined per cell for each segment on chromosome 4 and chromosome 18. Per segment the allele specific copy number was estimated by multiplying the estimated total copy number by the A and B allele frequency.

To define the copy number states, a second round of clustering of the cells was performed based on the integer copy number segmentation. Cells with hamming distance of zero were grouped to form the copy number states. Copy number states were sorted by the number of cells associated with the state, which ranges from 395 cells in copy number state 1 and 2 cells in copy number state 52. Copy number states with fewer than 2 cells were discarded. The segmented copy number calls along with the diplotype specific segmented copy number calls for chromosome 4 and 18 for each cell individually gives rise to the copy number state matrix.

#### Copy number tree inference

To extract a copy number tree we first infer a directed graph from the single cell copy number state matrix. Every node in the graph represents a single copy number state at one point in time. To incorporate a time axis in the graph, every copy number state is represented by one node for every time point a copy number state has been measured. When a copy number state of a particular clone is missing, we interpolate its abundance using linear interpolation. In this directed graph, every edge represents a copy number change and the weight of the edge represents the amount of edits between two nodes. Edges are pruned if they are biologically not plausible, e.g., in case they connect nodes with zero copies to a higher copy number or if they connect nodes in opposite temporal direction. A zero-weight edge is added between temporally adjacent nodes representing the same copy number state. An artificial root node is added to the graph wherein all segments are set to a diploid copy number state. From the resulting graph, an arborescence is extracted by using Edmonds algorithm, resulting in a copy number tree.

#### Copy number tree plotting

Copy number trees are plotted using a novel visualization, developed for this purpose, called ToverBoom. In Toverboom, each node represents a copy number state and the width of each node represents the relative amount of cells in the copy number state. Each branch represents a transition to another copy number state. The width of the nodes is smoothed using cubic interpolation. Lineage barcodes for each single cell were extracted from the single cell whole genome sequencing libraries. The lineage abundance was extracted from the bulk barcode sequencing libraries. Within one copy number state the relative abundance of every associated lineage barcode is calculated and projected on the lineage tree using a stacked area chart, where the area reflects the relative abundance of the lineage barcode within the associated copy number clone. The copy number tree inference and plotting code is available at https://github.com/BuysDB/ToverBoom.

#### Somatic single nucleotide variants detection

Variants were jointly called on 7841 cells derived from the three quadruple mutant replicates, and two other replicates (one single mutant and one double mutant which both serve as a normal control). All cells are descendants from the same donor.

Basecalling phred scores of the single cell bam files were recalibrated using GATK base quality score recalibration. All variants detected using the GATK HaplotypeCaller in the (Wildtype/P11N) bulk library and variants detected by Mutect2 in any of the bulk samples were supplied as known variation to be masked during covariate analysis. Candidate sSNVs were jointly called using BCFtools 1.9-174^42^ on a bam file containing all cells, and a threshold was set on the QUAL column for a phred score of at least 30.

To remove technical artifacts and germline variation only sSNVs uniquely detected in the quadruple mutant cells were kept, while sSNVs detected in single cells from the normal control samples were dropped. Furthermore, sSNVs detectable in the (Wildtype/P11N) bulk library with more than one read were dropped.

Haplotype phasing was performed using a strategy adapted from Bohrson et al.^32^ Briefly, for each sSNV phased heterozygous single nucleotide germline variants (gSNV) were determined in the (Wildtype/P11N) library. For the sSNVs with at least one phased gSNV, it was determined if phasing between the heterozygous gSNV and the sSNV is concordant in at least 95% of all cells, otherwise the sSNV was discarded. Molecules containing the sSNV are used as evidence indicative of presence of the sSNV. Absence of the sSNV is inferred when a cell has a molecule containing both the phased gSNV allele and the reference allele at the sSNV locus.

The sSNVs were further filtered by a random forest classifier trained on the 198150 sSNVs detected in bulk using Mutect2 as the ground truth. The features consisted of all the columns generated by the GATK variant caller, of which ReadPosRankSum and BaseQRankSum were most informative for classification. These features were appended with the following: the number of reads carrying the alternative base, the mean base quality of the alternative base in the single cell data, the mean number of gSNVs overlapping with reads containing the alternative allele and the mean number of gSNVs overlapping with reads containing the reference allele and the complexity of the reference sequence in a 75bp, 150bp, 300bp, 500bp and 1kb window, encoded by counting the number of unique 5bp and 7bp k-mers. Final classification of the candidate variants was performed using leave one out cross-validation. The classifier used is a sklearn random forest classifier with 100 trees and class balancing weights enabled. Finally, all selected variants were inspected in a genome browser (IGV). A few variants were removed upon manual inspection.

#### Somatic single nucleotide variant imputation

Genotypes of all quadruple mutant single cells to which a copy number state could be assigned are inferred and imputed using a Bayesian inference algorithm, SiCloneFit.^43^ The imputation allows for clustering of the single cell SNV genotypes. Only variants which were present in at least 2 cells and only cells with at least 4 sSNVs were used for the imputation. Expected false negative and false positive rates of the sSNV measurements are set at 0.001 and 0.0001, respectively. SiCLoneFit utilizes Gibbs sampling of the posterior distribution of measured SNVs to infer tumor clones, phylogeny, and genotype in each tumor clone. The missing sSNV measurements are imputed according to their genotype in the assigned clone. The imputed sSNVs were then combined with the measured sSNVs and clustered and plotted (Figure 4B). The imputation and visualization tools are available at https://github.com/zztin/siCloneFitIO. To test the accuracy of the imputation we performed 10-fold cross-validation by leaving out a fold of 10% of known sSNV calls. The estimated accuracy is approximately 0.86.

#### Neutral drift simulations

To investigate neutral drift, we performed *in silico* stochastic simulations. To this end, three parameters were defined: the replication rate (rr) (number of cell divisions per h) of the organoids, the number of cells starting the population (sp) and the number of cells that were retained when passaging the culture (bottle neck size (bns)). The simulation was then executed as follows. Every cell in the starting population is considered a unique clone, every h every cell belonging to a certain clone has a certain probability to proliferate (rr). When a cell proliferates the number of cells belonging to that clone increases by 1. After 168 h (1 week) bns cell are randomly selected and allowed to restart the culture. This process then continues for 25 weeks. Finally, the Shannon’s entropy of the clones in the culture was analyzed to estimate the clonal dynamics.

### Quantification and statistical analysis

#### MSKCC colorectal cancer dataset analyses

Data from colorectal cancer samples generated using the MSKCC impact sequencing panel was obtained through the European variation archive, accession PRJEB23844. From this only micro satellite stable samples were selected for which clinical follow-up data was available, resulting in a total of 1027 samples. To test for independence of the chromosome 18 and chromosome 4 copy ratios, we performed 100.000 resampling’s of the chromosome 4 copy ratio relative to the chromosome 18 copy ratio across all patients. We then compared the number of tumors in which the chromosome 4 ratio was lower than the chromosome 18 ratio in the resampling’s to the number of tumors in which the chromosome 4 ratio was lower than the chromosome 18 ratio in the actual tumors. The p value for the permutation test is the fraction of resampling’s in which there was a higher fraction of tumors with chromosome 4 ratio lower than the chromosome 18 ratio than in the actual samples.

#### Conditional chromosomal aberration analysis

Contigs (entire chromosomes, except chromosome 8 which was split into 8p and 8q) were filtered on having an average absolute log copy ratio of > 0.4. For these contigs the absolute correlations of the log copy ratio for all combinations were calculated. For each combination of contigs present in at least 30 samples a Cox regression model was created in which the hazard ratio for tumors harboring both the priming and the conditional event was compared to tumors harboring only the priming event. P values were corrected using Benjamini Hochberg p value correction.

## Data Availability

• The accession numbers for the datasets reported in this study are available on SRA: Bio project id: PRJNA645018. • All original code has been deposited. The copy number tree inference and plotting code is available at https://github.com/BuysDB/ToverBoom the imputation generation code at https://github.com/zztin/siCloneFitIO and scripts at https://github.com/BuysDB/TumorEvolutionReconstruction. • Any additional information required to reanalyze the data reported in this paper is available from the lead contact upon request.

## Supplemental information

Document S1. Figures S1–S7

Table S1. Primers used for viral barcode PCR, related to STAR Methods

Document S2. Article plus supplemental information
