# Supplementary material for: Integration of multiple lineage measurements from the same cell reconstructs parallel tumor evolution
Source: Cell Genom. 2022 Feb 9;2(2):100096. doi: 10.1016/j.xgen.2022.100096 (PMC9903660; doi:10.1016/j.xgen.2022.100096)
Supplement: Document S1. Figures S1–S7 [file mmc1.pdf]

Cell Genomics, Volume *2*

Supplemental information

Integration of multiple lineage measurements from the same cell reconstructs parallel tumor evolution

Lennart Kester, Buys de Barbanson, Anna Lyubimova, Li-Ting Chen, Valérie van der Schrier, Anna Alemany, Dylan Mooijman, Josi Peterson-Maduro, Jarno Drost, Jeroen de Ridder, and Alexander van Oudenaarden

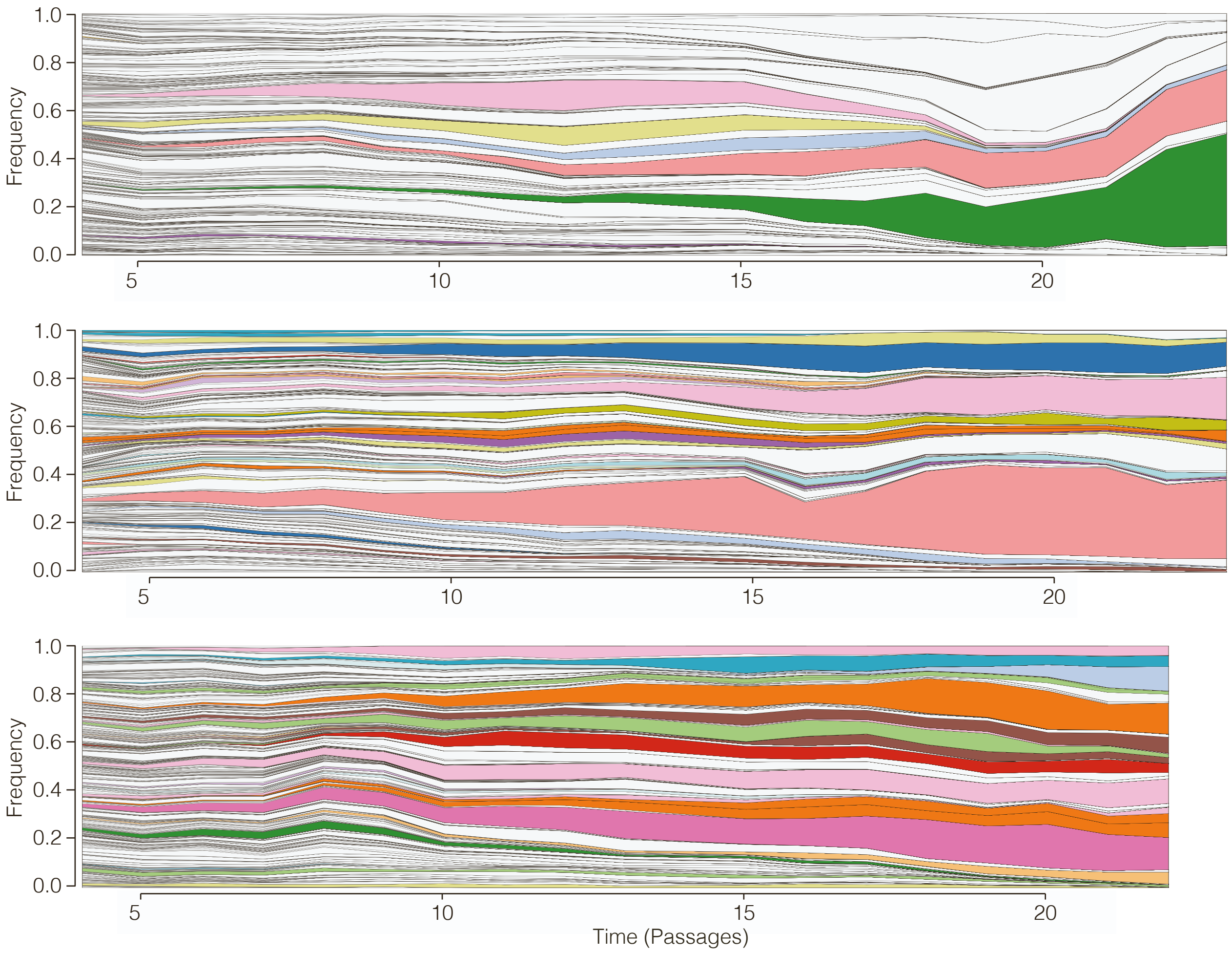

FIGURE S1, related to FIGURE 1. Clonal dynamics during *in vitro* evolution. (A-C) Relative frequency of observed viral lineage barcodes in replicates 1 through 3.

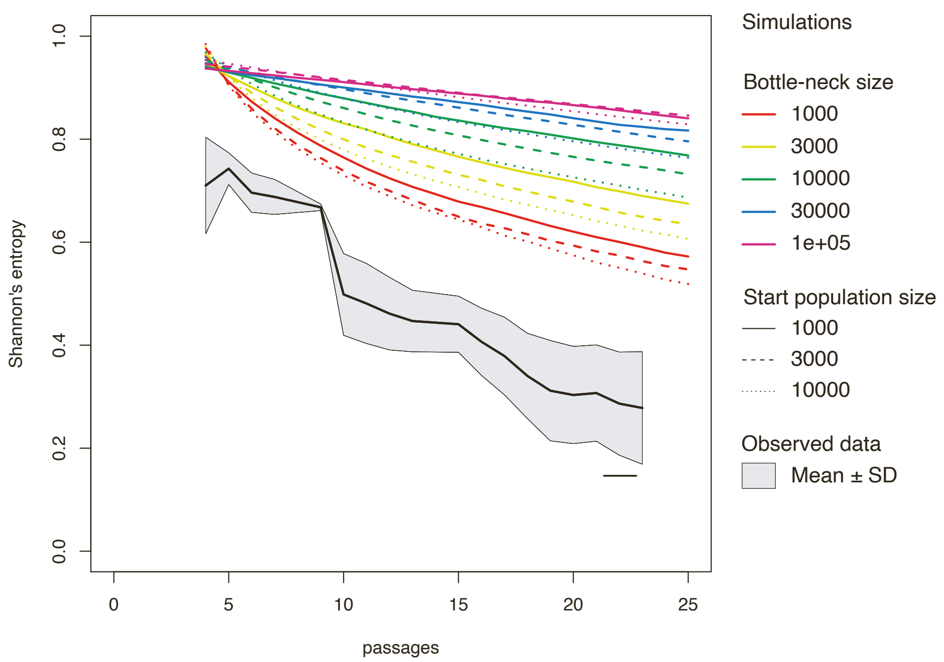

FIGURE S2, related to FIGURE 1. Simulation of clonal dynamics. Simulation of the Shannon entropy of the relative viral lineage barcode frequency as a function of time given a certain culture complexity at viral lineage introduction and a certain bottle neck size during the weekly passaging of the organoids (colored lines). Simulations assume there is no selection pressure on the culture and all cells have equal proliferative capacity. We calculated the Shannon's entropy of the clones as a measure for culture complexity. The replication rate was varied between 1/36, 1/48, 1/72 and 1/96 cell divisions per hour but there were no differences in entropy between the different proliferation rates, indicating that proliferation rate does not influence the clonal dynamics if it is assumed that all cells have the same proliferative potential. The entropy for the different simulations showed that the culture complexity is primarily depending on the bottle neck size, where a smaller bottle neck size shows a faster decrease in culture complexity, and to a lesser extend on the starting population size, where smaller starting sizes show a faster decrease in complexity.

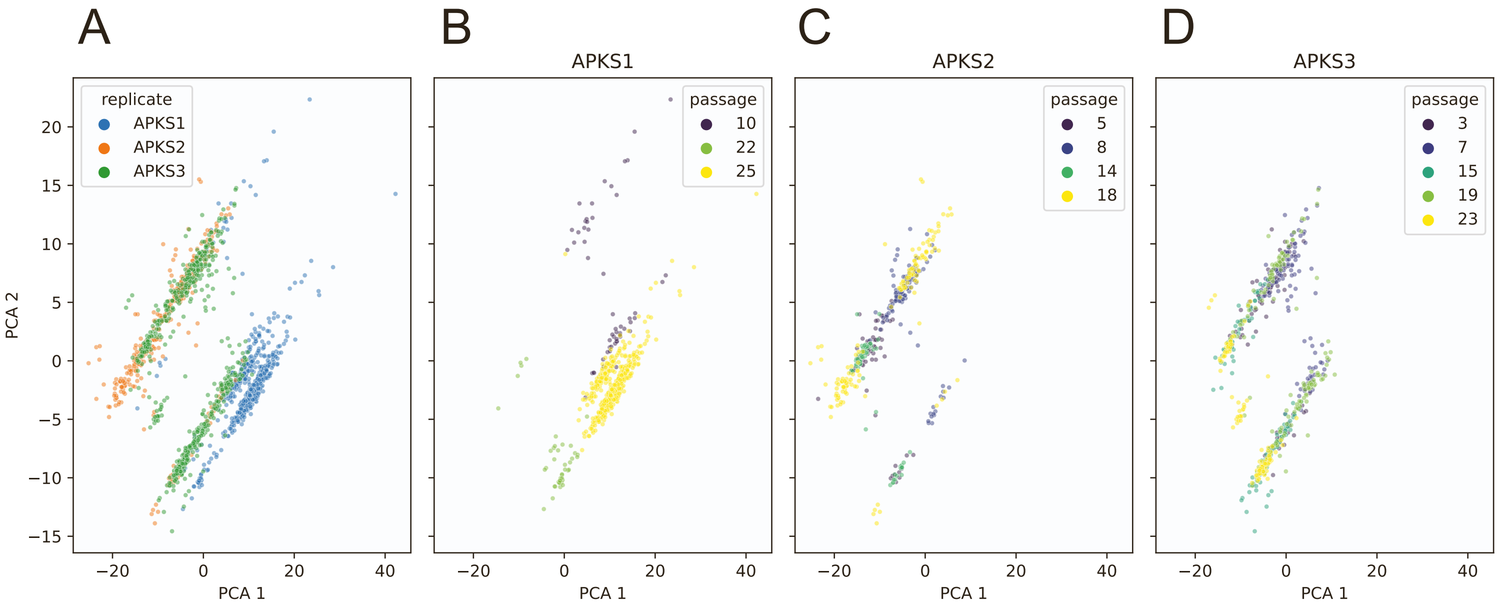

FIGURE S3, related to FIGURE 2. Quality control plots of the single cell copy number data by principal component analysis on the median normalized count matrix with allele specific counts on chromosome 4 and 18. Each dot corresponds to a single cell. (A) Cells are colored based on the replicate the cells belong to. (B-D) Cells of a single replicate are shown and the brightness of each dot indicates the passage (time-point).

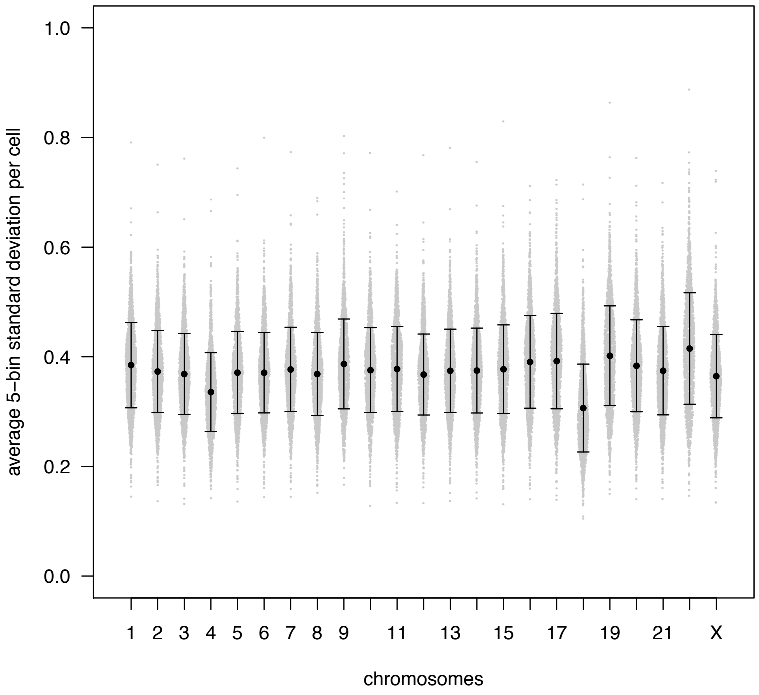

FIGURE S4, related to FIGURE 2. Variation in copy number measurement. Standard deviation of single cell CNV profiles, every dot indicates the average 5-bin standard deviation per chromosome for all single cells.

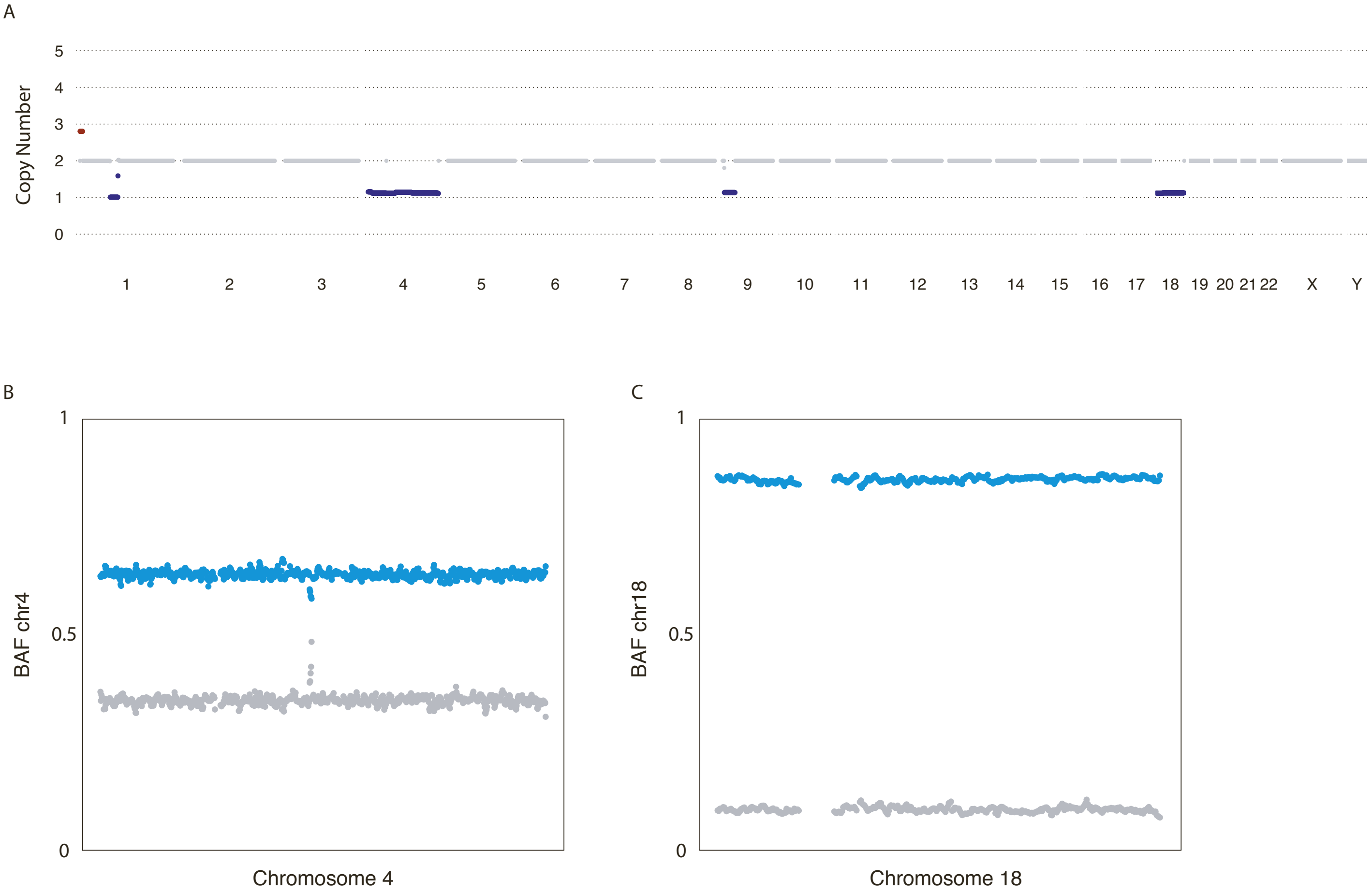

FIGURE S5, related to FIGURE 3. Bulk copy number profile (A): Bulk copy number profiles for replicate 1-3 at 23 passages. (B-C): B-allele frequencies in bins across chromosome 4 (B) and 18 (C), each bin contains 500 SNPs present in the Hapmap SNP project.

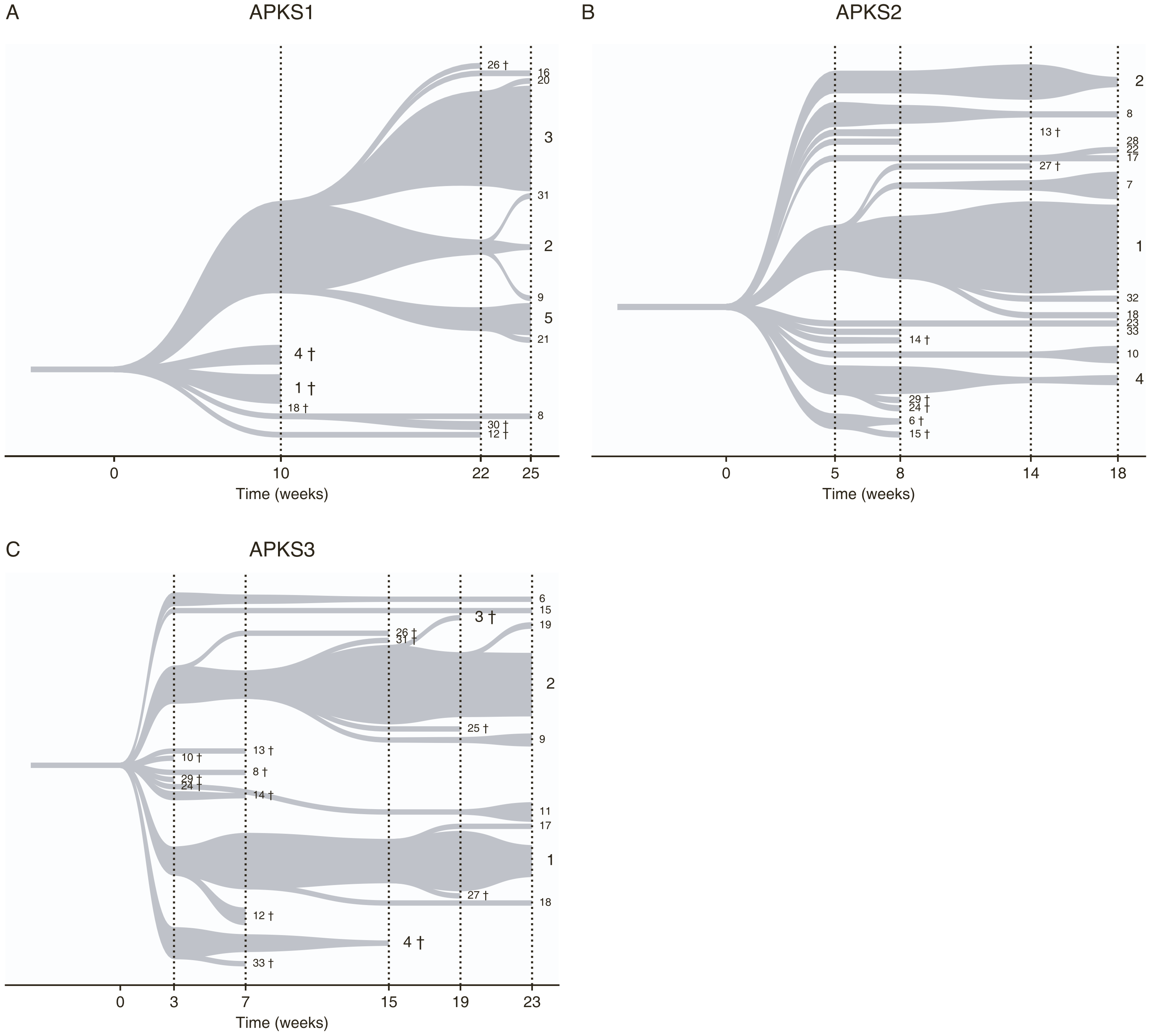

FIGURE S6, related to FIGURE 3. Clonal evolution trees constructed based on CNV states

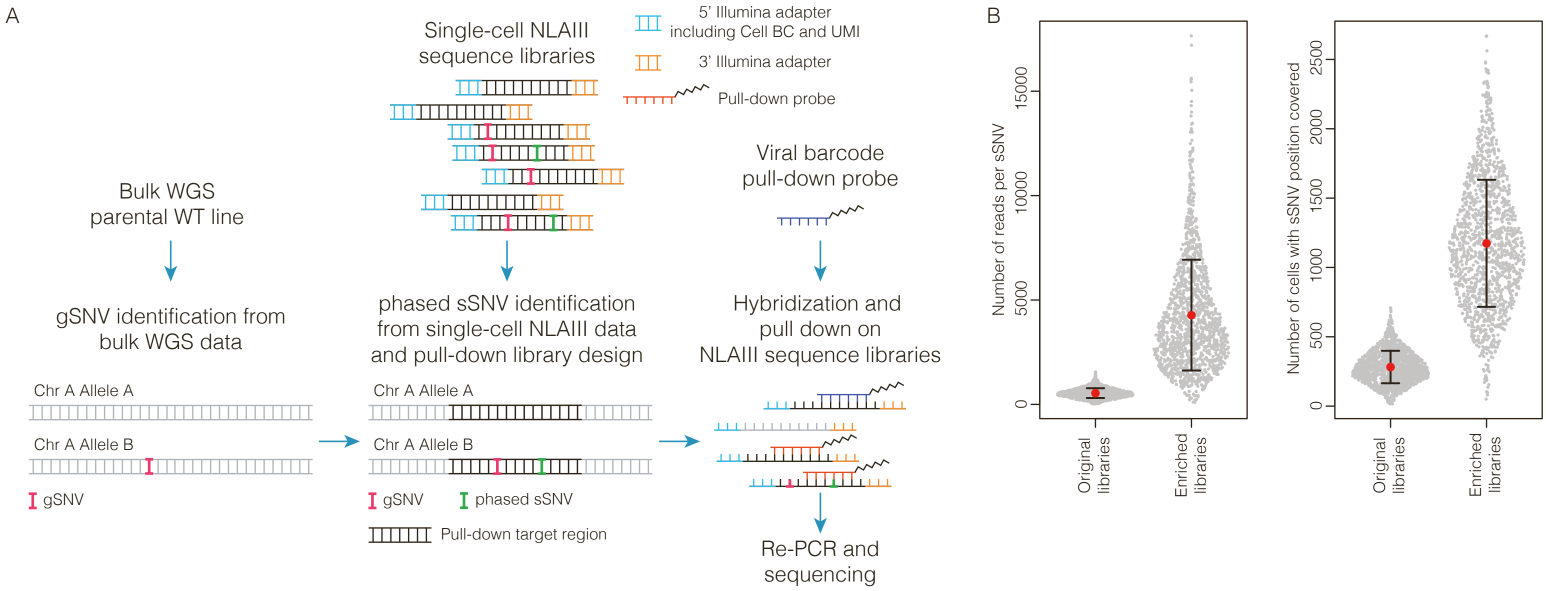

FIGURE S7, related to STAR methods. Enrichment of sSNVs positions from the single cell libraries (A) Enrichment strategy for the sSNV positions. Candidate sSNV positions were identified from a first round of sequencing of the single cell libraries. Anti-sense oligo's to the candidate positions were designed and hybridized to the single cell DNA libraries. Oligo's were pulled down from the mix and enriched libraries were sequenced. (B): Enrichment of candidate sSNV positions after pull-down enrichment.
